# Career Disruption and Employment Status of Korean Family Caregivers of Older Adults Using Home-Based Care

**DOI:** 10.3390/nursrep14030119

**Published:** 2024-06-27

**Authors:** Minah Lee

**Affiliations:** Department of Gerontology, Graduate School of East-West Medical Science, Kyung Hee University, Yongin 17104, Republic of Korea; minah.rhee@khu.ac.kr

**Keywords:** career disruption, employment status, home-based care, family caregiver, Republic of Korea

## Abstract

This study utilized nationally approved data from the 2022 Long-Term Care Survey of Korea to examine the factors associated with career disruptions and employment status among family caregivers of home-based care recipients. Descriptive statistics, chi-square tests, one-way ANOVA, and multinomial logistic regression analysis were employed to address the research questions. The results indicated that 19.39% of family caregivers of home-based care recipients experienced career disruptions due to informal caregiving. Demographic factors such as gender, age of family caregivers, and their relationship with care recipients predicted their employment status. Gender was a significant explanatory factor, as daughters/daughters-in-law were more likely to be in insecure employment positions than sons. Lower household income and older age were also associated with employment insecurity. Recommendations include coverage expansion, family support programs, and pension credit for family caregivers to meet the needs of care recipients and their families.

## 1. Introduction

The aging population in Korea is rapidly accelerating due to extended life expectancy and low birth rates. Prior to the introduction of the Long-term Care Insurance (hereafter LTCI) for older adults, as an independent scheme distinct from the pre-existing health care system to alleviate the costs of medical expenses and family care burden, aged care in Korea had been primarily carried out by families in the informal sector, except in cases where limited home-based care services targeting low-income older adults were provided as a part of means-tested programs of a few local governments. With the establishment and introduction of the LTCI for older adults in 2008, it became possible to discuss the defamilialization of aged care in Korea.

The LTCI scheme has expanded its coverage and benefits by introducing a cognitive assistance grade (grade 6), covering those diagnosed with low-grade dementia. Whereas grades 1–2 can utilize home-based and institutional care and grades 3–5 home-based care, grade 6 can only use day/night care benefits, offering relatively low care benefit cap (approximately 55.9% of grade 5). As a result of the government’s efforts, 10.9% of Koreans over 65 qualified for LTCI provisions as of late 2022 [1]. While the Korean LTCI provides two types of benefits—home-based care and institutional care—home-based benefits are gaining increasing importance, as aging in place has been prevalent. Among the six types of home-based benefits—home visit services, home bathing services, home nursing services, day/night care service, short-term respite care, and equipment provision service—the most essential is home visit services, where certified caregivers contracted through an agency visit users’ residences to provide personal care, such as helping with daily activities and household chores. While high grades with lower levels of activities of daily living and instrumental activities of daily living are more likely to use institutional care, relatively healthier older adults are the main beneficiaries of home care services. Among the Korean population aged over 65, 8.1% receive home visit services [2].

With the establishment of the LTCI and the emphasis on the importance of home-based care and aging in place, many studies have been conducted to verify the effects of home-based care services as formal care on the informal care and care burden of older adults’ families, reporting positive effects on reducing care stress and increasing social participation [3,4,5]. Despite existing research suggesting that Koreans’ attitudes toward aged care have already significantly defamilialized compared to other countries [6], even after the introduction of the formal care system of the LTCI, aged care in Korea is still largely provided by family members, especially by women [7]; 61.4% of the family caregiver participants of the 2022 LTC Survey were female [8].

Revisiting the role of the state and family with regard to the LTCI, familialism still plays an crucial role in aged care in Korea [9] even though the public sector shares the care burden—there are no options for cash benefits, no options for direct employment of care workers, and high levels of copayment (15% for home-based care, 20% for institutional care). Unlike other familialistic countries, such as Italy and Spain, Korea did not implement cash-for-care to avoid female family members bearing care responsibilities, as this may discourage them from participating in the labor market [10]. If families continue to take on caregiving roles predominantly, the familialization of aged care provided by female family members may be intensified due to social norms. Unlike European countries, LTCI in Korea does not permit the hiring of care workers in the market. This leads to a substantial caregiving responsibility continuing to be shouldered by family members, particularly daughters [8].

In particular, the burden of aged care on families persists despite the benefits of home care services. Unlike institutional care, which provides continuous 24 h care, home-based care has a limitation in usage, with a care benefit cap, and care is interrupted when the limit is reached. Any costs exceeding the monthly cap are fully borne by the individuals, leading to gaps in care when long-term care benefits are not utilized. Ultimately, the responsibility to close these gaps falls upon family caregivers. As of 2024, older adults classified under the most severe grade, grade 1, are allowed approximately 6 h of daily home-based care.

Given that home-based benefits cannot guarantee ongoing care, family members struggle to balance work and family. According to the 2022 Long-Term Care Survey, 19% of family respondents reported having experienced career disruptions due to caregiving responsibilities for their spouses, parents, or grandparents. Career disruption, defined as withdrawing from the labor market due to caregiving responsibilities, mostly indicates mothers leaving work due to factors related to maternity, such as pregnancy, childbirth, and childcare. In the Korean context, career disruptions often explain the low labor market participation of women. Although somewhat alleviated by the recent decline in marriage rates in Korea, a distinct M-shaped curve has been observed in women’s labor market participation: many Korean women withdraw from the labor market after marrying due to pregnancy, childbirth, and childcare responsibilities, then re-enter the labor market in their 40s after their children have grown [11,12]. However, after their childcare duties end, many adults also provide care for their older spouses and parents/parents-in-law. Academic interest in care responsibilities for spouses, parents/parents-in-law, or grandparents is increasing. Unlike childcare, aged care tends to be more prolonged and intensify over time as the physical and mental health of older adults deteriorates, requiring caregivers to become knowledgeable of disabilities or chronic diseases [13,14].

Several studies have focused on the relationship between family caregiving and labor market participation, claiming that informal care plays a crucial role in explaining employment. The results have been inconsistent, with some studies reporting a negative relationship between family caregiving and employment [15,16,17,18] and others finding no correlation [19,20]. In Korea and Japan, where female family members are considered primary caregivers, studies focus on the female workforce. It is claimed that the relationship between informal caregiving and labor supply was limited after the introduction of the LTCI both in Korea and Japan [3,21], citing inadequate benefits as a cause [3]. Similarly, it is suggested that social norms regarding intergenerational obligation associate negatively with female employment in Europe [22]. On the other hand, in a comparative study of Europe and Korea at the micro level, only in the Netherlands did being an informal caregiver associate with being employed [23]. In the Norwegian context, parental care has a negative impact on the employment trends in adult children who experienced their parents’ death [24], while being a daughter is an explanatory variable in having difficulties in combining work and care [25]. The age of caregivers, caregiving intensity, and the use of home-visit care were associated with the unemployment of family caregivers [25,26]. Studies have also shown variations in labor market participation based on primary caregiver status and caregiving intensity, presenting a dynamic relationship between informal care and employment status [27].

Thus, gender is another key to explaining how aged care and employment correlate. Several previous studies focus on women’s responsibilities to care for family members. When children are still in need of care, women can face a dual caregiving burden, having to care for their children and their parents (or in-laws) simultaneously. Recent research even suggests that a shift in the primary caregiver for parental care from daughters-in-law to daughters has been found, particularly when daughters are single [7,28]. Studies have also identified gender differences in caregiving experiences among spouses [29,30,31]. Research findings also indicate that the socialization of adult care has reduced the amount of time spent on care by females who provide care for older adult family members [32], suggesting a gender bias in aged care responsibilities among family members. Differences in the relation between family caregivers and care recipients have been mentioned in relation to caregivers’ employment status [33,34].

Factors related to caregiving intensity can also lead to work–family conflicts, which may affect employment status. Employed family members experience higher caregiving burdens compared to their unemployed counterparts [35]. The health status of care recipients and the level of independent activities performed by older family members were also identified as factors contributing to the employment of caregivers [30,35]. People providing high-intensity caregiving show a higher probability of early retirement before 65 compared to those who do not [36]. Additionally, children living with their parents are more likely to be unemployed [37], indicating that the intensity of care provided by primary caregivers may be an explanatory variable.

The family caregivers who are responsible for aged care experience stress and care burden, both of which tend to decrease with increased usage of formal care [8,38]. In particular, family members of home-based care recipients feel a greater care burden compared to families of institutional care benefit recipients, indicating that formal care complements rather than fully replaces informal care.

Despite the importance of aged care by families in the context of emphasizing aging in place, studies focusing on the employment status of family caregivers who care for older adults in Korea have not been prevalent. Thus, this study aims to analyze the re-entry of Korean families into the labor market after experiencing career disruption due to care responsibilities.

Based on the literature reviewed above, this study aims to verify the explanatory variables of career disruption and employment status among families providing aged care. Although labor market exits due to care burdens have been predominant in Korea for a substantial time, research on formal care users, their families, and their employment status has been lacking. Since career disruptions and employment status have mainly been discussed in relation to maternity or the middle-aged labor market transition, further research is required on how informal care responsibilities relates to family caregivers’ labor market experiences. Is being an informal caregiver associated with career disruption? Does employment status differ due to the characteristics of care? Does gender determine family caregivers’ experiences with regard to employment? To answer these questions, this study aims to investigate factors related to career disruption and employment status among families receiving home-based care benefits, utilizing secondary data from the 2022 Long-Term Care Survey.

## 2. Materials and Methods

### 2.1. Sample and Data

The Long-Term Care Survey was conducted to understand the needs of the interested parties and the state of the LTCI. The Korea Institute For Health And Social Affairs conducts the survey as part of the Nationally Approved Statistics (No. 117104) program ordered by the Minister of Health and Welfare; the survey has been undertaken tri-annually based on Article 6-2 of the Long-Term Care Insurance Act since 2019 and is exempt from ethical approval and review.

It includes three domains of data on long-term care workers, long-term care institutions, and long-term care beneficiaries and their families. In this study, the third dataset is used, which includes LTC beneficiaries and family caregivers. The survey targeted the whole population utilizing long-term care benefits and their family caregivers, selected using the stratified systematic method to reflect the characteristics of beneficiaries approved by National Health Insurance Service, the public organization that operates the LTCI in Korea. Among them, there were 3371 dyads of older adults and family caregivers, while 1042 older adults participated without a family caregiver, using online and face-to-face questionnaires [8].

### 2.2. Measurements

Personal and care-related factors of home visit care recipients and family caregivers are included as independent variables based on the literature reviewed in the previous section. Two multinomial logistic regression models were designed to explore employment status after career disruption. The dependent variable in the first model is career disruption and re-entry, whereas the dependent variable in the second model is the employment status of those who re-entered the labor market. An overview of variables in the models and response options for questions and operational definitions are shown in Table 1.

### 2.3. Data Analysis

To verify factors associated with career disruptions and employment status of home-based care recipients’ families, descriptive statistics, one-way analysis of variance (one-way ANOVA), and logistic regression analysis were conducted. Stata version 17 was used to analyze the study model.

## 3. Results

### 3.1. Descriptive Statistics

#### 3.1.1. Descriptive Statistics of Care Recipients

First, frequency analysis was conducted to identify the characteristics of care recipients. While approximately half of them were in their 80s, 36.46% had spouses, with an average of 2.09 family members living with them. Family members caring for recipients in their sixties had more career disruptions than other age groups. Approximately one-third (33.24%) were living alone, while approximately 65.75% were living with spouses, children, or grandchildren. The average number of diagnosed diseases among the recipients was positively associated with career disruption. On average, recipients were diagnosed with 4.96 diseases, with 52.63% being diagnosed with dementia. The inability to move independently was found among 18.37% of respondents, while 48.22% were grade 4. On average, they received 1648.17 min of home visit care in the previous month. The highest proportion of respondents lived in rural areas (36.12%). Detailed frequency analysis results regarding characteristics of care recipients with and without experiences of career disruptions among family caregivers are shown in Table 2.

#### 3.1.2. Descriptive Statistics of Family Caregivers (n = 2068)

Next, the characteristics of family respondents were examined. A total of 2068 primary caregivers were explored. First, women accounted for 63.01% of family caregivers, and the average age of family caregivers was 60.83. The perceived care burden from four different types of support averaged 2.69, and the average caregiving intensity was 3.80, with an average out-of-pocket expense of KRW 138,180. Those cohabitating with the caregiver accounted for 51.16%, and households with a monthly income of less than KRW 1 million comprised the largest portion at 25.82%. The frequency analysis results are presented in Table 3.

### 3.2. Care Recipients’ Characteristics and Experiences of Career Disruption

One-way ANOVA and chi-square tests were conducted to find differences based on whether family member caregivers experienced career disruptions. However, because the question in the LTC survey asking, “Had the care recipient’s spouse or one of the immediate family members quit their job due to care responsibility?” does not exclusively target the primary caregivers themselves, the career disruption experience within the family reported by family respondents may not necessarily be that of the primary caregivers. Therefore, only variables related to care recipients were considered, excluding caregiver variables, to avoid the confusion of subjects.

The analysis revealed that differences in family career disruption status were not statistically significant across gender, marital status, home-based care usage time, and region type. However, among the relatively younger care recipients, career disruption of the family member caregiver was more frequently observed. Moreover, it appeared that career disruption was more common in multi-generational households with children or grandchildren compared to single-person or older-adult-couple households, indicating that adult children living with and caring for parents were more likely to experience career disruptions. Additionally, households that experienced career disruptions tended to have more family members. The number of diagnosed diseases was also higher in the group with career disruption experience, with dementia diagnoses being more prevalent in this group as well. The range of activities that can be performed without assistance and LTC grade indicated a correlation between the health of older adults and career disruption. Furthermore, living with the primary caregiver was associated with a higher frequency of career disruption, suggesting that those who experienced career disruption were more likely to be live-in family caregivers. Table 4 presents the detailed results.

### 3.3. Employment Status and Family Caregivers’ Characteristics

Before proceeding with multinomial logistic regression analysis, one-way ANOVA and chi-square tests were conducted to examine the relationship between employment status and the characteristics of care recipients and family caregivers. The results show that all variables related to care recipients, except for age and out-of-pocket expenses, were associated with employment status. Among the unpaid family caregivers, females and self-employed family members were overrepresented. Care recipients with spouses were most commonly found within the unemployed caregiver group. When the caregiver was a full-time worker, the care recipient was most likely to be living alone, while in cases where the caregiver was unemployed, the care recipient was most commonly residing with their children. The average number of family members was highest when the care recipient’s caregiver worked as an unpaid family member, and the average number of diagnosed diseases was highest when the care recipient was unemployed. Dementia was also most frequently found in the unpaid family workers group. Grade 1 recipients were more likely to have unemployed caregivers. Unpaid family workers used home-based care for the longest time, and employment status and region were also correlated. Detailed results are presented in Table 5.

### 3.4. Factors Affecting Employment Types of Family Caregivers of Home-Based Care Recipients

Multinomial logistic regression analysis was conducted to identify factors explaining the current employment status of primary caregivers within families. Only factors identified as related to employment status through one-way ANOVA and chi-square tests were included in the model, aiming to examine the impact of informal caregiving within families on the employment status of primary caregivers.

The analysis revealed that variables related to care recipients were not significant across all groups. Instead, the determinants of primary caregivers’ employment status were found to be demographic factors, caregiving intensity-related factors, and the relation with the care recipient rather than the care needs or informal resources of the care recipients.

Compared to full-time employment—the most secure employment status—part-time or temporary work was associated with older age (RRR = 1.054), lower household income (RRR = 0.626), and a higher likelihood of the primary caregiver being a daughter or daughter-in-law rather than a son. Regarding the self-employment group, only the age of caregivers and care burden were significant. Older individuals (RRR = 1.070) or those perceiving less care burden (RRR = 0.841) were more likely to be self-employed than employed full-time. The relation with the care recipient variable was not significant.

For unpaid family workers, only household income and relation variables were significant. They were more likely to have lower income (RRR = 0.808) compared to full-time employees and were more likely to assist with family tasks unpaid if they were the daughter (RRR = 2.739), daughter-in-law (RRR = 6.240), or other family members (grandchildren, sons-in-law, siblings, etc.) (RRR = 4.080) of the care recipient rather than the son.

Lastly, unemployed caregivers were significantly associated with age, household income, and relation with the care recipient. Older caregivers (RRR = 1.096) with lower household income (RRR = 0.666) were more likely to be unemployed compared to full-time workers. They were also more likely to be daughters (RRR = 2.439), daughters-in-law (RRR = 3.880), or other family members (RRR = 3.112) rather than sons. These results are presented in Table 6.

## 4. Discussion

Even after the introduction of the LTCI, family caregivers of home-based care recipients continue to provide informal care, leading some to quit their jobs due to caregiving responsibilities. This study aims to investigate differences between groups based on whether family caregivers experienced career disruption and analyze the factors influencing employment status among family caregivers of home-based care recipients.

Out of a total of 2068 recipients of home care benefits, 19.39% had immediate family caregivers who experienced career disruption due to care responsibilities. Through one-way ANOVA and chi-square tests conducted to understand the characteristics related to career disruptions, differences among groups were explained by factors such as the care recipients’ age, household type, number of diagnosed diseases, presence of dementia, and cohabitation with the primary caregiver.

Furthermore, the study aimed to analyze differences in the employment status of primary caregivers based on factors related to informal resources, demographic characteristics of primary caregivers, caregiving provision, and relation to care recipients. The one-way ANOVA and chi-square tests showed that, except for the age of care recipients and out-of-pocket expenses, all other variables showed significant associations with the employment status of primary caregivers.

Lastly, multinomial logistic regression analysis was conducted using all variables previously identified as relevant to explore the impact of informal caregiving-related factors on the current employment status of primary caregivers. The analysis revealed that the care needs and informal resources of older adults did not show statistically significant associations with employment status across all categories. Instead, primary caregiver age, household income, and relation with the care recipient were the only consistent factors showing significant influence.

Based on the research findings above, several conclusions can be drawn. First, the employment status of primary caregivers appears to be influenced more by demographic factors than by care recipients’ needs, informal resources, or care burden. Although not included in this paper, when only variables related to the care recipient were considered, they were significant predictors of employment status. However, when demographic and relationship variables of primary caregivers were included, their significance diminished. The average age of primary caregivers in this study (60.83) is close to the legal retirement age, which is 60 in Korea, suggesting that the challenges of employment and job retention for middle-aged and older adults significantly influenced the results.

Second, compared to the relatively secure full-time employment group, the vulnerability of the other comparison groups became apparent. Specifically, as primary caregivers’ age increased, they were more likely to be temporary or part-time workers, self-employed, or unemployed. Similarly, lower household income was associated with a higher likelihood of engaging in temporary or part-time work, providing unpaid family work, or being unemployed.

Lastly, significant gender differences were evident. Even among caregivers who had experienced career disruptions, women were more prevalent. Furthermore, primary caregivers with unstable employment status, such as temporary or part-time workers, unpaid family workers, or unemployed individuals, were more likely to be female members within the family, particularly daughters and daughters-in-law, compared to sons. Particularly noteworthy is that, among caregivers with full-time employment, daughters-in-law (RRR = 6.240) and daughters (RRR = 2.739) are more likely to be unpaid family workers compared to sons. This suggests that daughters and daughters-in-law are more likely to provide unpaid caregiving assistance and support family-owned businesses within the family, as opposed to being engaged in paid employment.

Based on the findings, the following recommendations are proposed. First, policies aimed at supporting the vulnerable employment status of female caregivers within families are needed. Compared to male caregivers, daughters and daughters-in-law are more likely to take on informal caregiving responsibilities while simultaneously being at a higher risk of unstable employment or even unemployment. Even without cash benefits in Korean LTC, care responsibilities still hinder women’s employment. Due to their insecure employment status, they earn lower incomes and are more likely to face vulnerability in securing retirement income in the future, especially considering their average age (57.1). Measures such as shortened or flexible working hours and expanded paid caregiving leave for middle-aged and older caregivers must be implemented to ensure their long-term employment stability.

Secondly, to reduce career disruptions due to caregiving responsibilities, benefits for dementia patients must be expanded. In this study, a higher prevalence of dementia was observed among those who experienced career disruptions. Even in the mild severity grades, the number of diagnosed dementia patients was over four times higher than those without dementia. However, the cognitive assistance grade (grade 6), which encompasses mild dementia patients, is only allowed the use of day and night care services, posing a risk factor for employment insecurity among family caregivers. Therefore, policy support is needed to expand benefits for dementia patients and their families to reduce career disruptions.

Thirdly, care should be reorganized in a more comprehensive way that integrates all demographics, including children and adults, within the life course of labor and caregiving. Care for older adults, especially, is a complex process that involves not only main caregivers but also multiple family members and economic, temporal, and emotional resources over an extended period of time [39]. As care responsibilities can be more burdensome for those who are vulnerable, various services for family caregivers need to be provided. While paid caregiving leave and flexible working hours are required for employed caregivers, supports such as counselling, health check-ups, and leisure activities can be considered for unemployed caregivers.

Lastly, to compensate for career disruptions experienced by family caregivers, introducing pension credits should be considered. As being female or older may result in a more insecure employment status, there is a need to establish measures for integrating caregiving periods into social security systems. Given that employment status may determine pension benefits, career disruptions and precarious employment status due to caregiving responsibilities can lead to poverty in later life. International examples can be referenced. For example, family caregivers of the LTC recipients in Germany and Carer’s Allowance recipients in the UK are entitled to pension credits for their care responsibilities [40]. However, the National Pension Service in Korea provides pension credits only for childbirth, unemployment, and military service. This coverage should be expanded to include family caregivers of LTC recipients to increase the capacity to prevent old-age poverty among vulnerable family caregivers.

### Limitations

This study aimed to analyze employment status and the experience of career disruptions among recipients of home care benefits and their families. However, those who experienced career disruptions may not be the respondents themselves, preventing this research from providing an integrated analysis of these two factors. Additionally, despite the importance of considering changes over time in career disruptions or employment status, which could provide a deeper understanding, this study was limited to cross-sectional analysis lacking time-specific variables. Further research is needed to utilize longitudinal data to explore the needs of long-term care recipients and support for balancing work and care, which can serve as foundational information for future studies.

## 5. Conclusions

This study utilized the 2022 Long-Term Care Survey approved by Statistics Korea to investigate the factors affecting career disruptions and employment status among family caregivers of home-based care beneficiaries. Due to the inherent limitations of public home-based care services, families still engage in informal care, which poses risks to the employment stability of family caregivers. Among the total study participants, 19.39% experienced a career disruption due to care responsibilities. It was also found that the demographic and socioeconomic factors of caregivers, as well as their relation with the care recipients, influenced their employment status. Based on the findings, this study suggests policy support measures and calls for more in-depth follow-up research related to informal care and the employment status of family caregivers.

## Figures and Tables

**Table 1:** Variables in the study.

Response Options for Questions/Operational Definitions		
Care recipients	Gender	Male (0), female (1)
	Age	<64 (1)~<90 (7)
	Marital status	Without spouse (0), with spouse (1)
	Family type	Living alone (0), W/spouse (1), W/children (2), W/child + grandchild (3), others (4)
	Home care usage	Used home visit minutes last month
	Region	Rural (0), medium and small city (1, with population less than 500,000), large-sized city (2, with population over 500,000)
	LTC grade	1~5, and 6 (Cognitive Assistance Grade) with smaller numbers indicating higher frailty
	Dementia diagnosed	Yes (1), No (0)
	Out-of-pocket money	Costs of co-payments for home-based care services last month (KRW 1000)
Family caregivers	Employment status	Full-time (1), part-time and temporary (2), self-employed (3), unpaid family work (4)
	Career disruption	Whether the care recipient’s spouse or other immediate family member quit their job due to care responsibility Yes (1), No (0)
	Age of caregiver	Continuous
	Household income	KRW < 1 million (1) ~ > KRW 6 million (7)
	Cohabitation with care recipients	Yes (1), no (0)
	Care Burden	Average emotional, daily support, household chores, and physical care burden Not at all (1)~Very much burdened (5)
	Caregiving intensity	Total frequency of emotional, daily support, household chores, and physical care none (0)~three time a week (5)
	Relation with care recipients	Spouse (1), Son (2), Daughter (3), Daughter-in-law (4), Others (5)

**Table 2:** Characteristics of care recipients (n = 2068).

Variables	Values	Total	
		Freq. (n)/Mean	%/SD
Gender	Male	579	28.0
	Female	1489	72.0
Age	<64	36	1.74
	65~69	80	3.87
	70~74	143	6.91
	75~79	284	13.73
	80~84	540	26.11
	85~90	601	29.06
	90<	384	18.57
Marital status	Without spouse	1374	66.44
	With spouse	694	33.56
Family type	Living alone	707	34.19
	W/spouse	495	23.94
	W/children	727	35.15
	W/child + grandchild	109	5.27
	others	30	1.45
Number of family members		2.13	1.18
Number of diagnosed diseases		4.90	2.12
Dementia diagnosed	Yes	1097	53.05
	No	971	46.95
LTC grade	Grade 1	81	3.92
	Grade 2	156	7.54
	Grade 3	539	26.06
	Grade 4	1004	48.55
	Grade 5	268	12.96
	Grade 6	20	0.97
Used home visit minutes last month		1635.99	1663.12
Region	Large-sized city	671	32.45
	Medium and small city	634	30.66
	Rural area	763	36.9
Out-of-pocket expenses (KRW 1000)		138.18	108.33
Cohabitation with primary caregiver	Yes	1058	51.16
	No	1010	48.84

**Table 3:** Characteristics of family caregivers (n = 2068).

Variables	Values	Total	
		Freq. (n)/Mean	%/SD
Gender	Male	765	36.99
	Female	1303	63.01
Age		60.83	11.09
Care burden		2.69	1.02
Caregiving Intensity		3.80	1.18
Household income (KRW 100,000)	10>	534	25.82
	10–20	435	21.03
	20–30	403	19.49
	30–40	293	14.17
	40–50	197	9.53
	50–60	109	5.27
	60<	97	4.69
Relation with care recipients	Spouse	395	19.1
	Son	776	37.52
	Daughter	232	11.22
	Daughter-in-law	577	27.9
	Others	88	4.26

**Table 4:** Career disruption and care recipients’ characteristics (n = 2068).

Variables	Values		With Career Disruption		Without Career Disruption		Pearson’s Chi^2^/ Bartlett’s Chi^2^
			Freq. (n)/Mean	%/SD	Freq. (n)/Mean	%/SD	
Gender	Female		112	27.93	467	28.01	0.973 **
	Male		289	72.07	1200	71.99	
Age	<64		10	2.49	26	1.56	17.8889 **
	65~69		27	6.73	53	3.18	
	70~74		33	8.23	110	6.60	
	75~79		61	15.21	223	13.38	
	80~84		94	23.44	446	26.75	
	85~90		113	28.18	488	29.27	
	90<		63	15.71	321	19.26	
Marital status	Without spouse		265	66.08	1109	66.53	0.0283
	With spouse		136	33.92	558	33.47	
Family type	Living alone		99	24.69	608	36.47	46.4911 ***
	W/spouse		75	18.7	420	25.19	
	W/children		192	47.88	535	32.09	
	W/child + grandchild		30	7.48	79	4.74	
	others		5	1.25	25	1.5	
Number of family members			2.41	1.28	2.07	1.15	7.6919 **
Number of diagnosed diseases			5.32	2.32	4.79	2.06	9.3815 **
Dementia	Yes		240	59.85	857	51.41	9.2416 **
	No		161	40.15	810	48.59	
LTC grade	Grade 1		29	7.23	52	3.12	45.2698 ***
	Grade 2		44	10.97	112	6.72	
	Grade 3		130	32.42	409	24.54	
	Grade 4		158	39.4	846	50.75	
	Grade 5		35	8.73	233	13.98	
	Grade 6		5	1.25	15	0.90	
Home care usage			1650.47	1702.05	1632.50	1654.12	0.1027
Living with family caregivers		No	148	14.65	862	85.35	28.3439 ***
		Yes	253	23.91	805	76.09	
Region	Large city		141	35.16	530	31.79	0.5321
	Medium and small city		120	29.93	514	30.83	
	Rural area		140	34.91	623	37.37	

** *p* < 0.01; *** *p* < 0.001.

**Table 5:** Employment status and family caregivers’ characteristics (n = 2068).

Variables	Values	Full-Time		Part-Time and Temporary		Self-Employed		Unpaid Family Work		Unemployed		Pearson’s Chi^2^/ Bartlett’s Chi^2^
		n./Mean	%/SD	n./Mean	%/SD	n./Mean	%/SD	n./Mean	%/SD	n./Mean	%/SD	
Gender (c.p.)	Female	440	75.34	225	66.18	258	78.9	44	83.02	522	68.32	24.9886 ***
	Male	144	24.66	115	33.82	69	21.1	9	16.98	242	31.68	
Age	<64	13	2.23	2	0.59	2	0.61	0	0.00	19	2.49	34.9213
	65~69	22	3.77	15	4.41	12	3.67	0	0.00	31	4.06	
	70~74	33	3.77	28	4.41	15	3.67	4	0.00	63	4.06	
	75~79	83	14.21	50	14.71	34	10.40	6	11.32	111	14.53	
	80~84	169	28.94	82	24.12	95	29.05	13	24.53	181	23.69	
	85~90	173	29.62	98	28.82	105	32.11	15	28.30	210	27.49	
	90<	91	15.58	65	19.12	64	19.57	15	28.30	149	19.50	
Marital status	Without spouse	447	76.54	216	63.53	236	72.17	41	77.36	434.00	56.81	67.4676 ***
	With spouse	137	23.46	124	36.47	91	27.83	12	22.64	330.00	43.19	
Family type	Living alone	249	42.64	101	29.71	134	40.98	17	32.08	206	26.96	99.5554 ***
	W/spouse	86	14.73	86	25.29	58	17.74	7	13.21	258.00	33.77	
	W/children	208	35.62	131	38.53	111	33.94	25	47.17	252	32.98	
	W/child + grandchild	35	5.99	17	5	20	6.12	2	3.77	35	4.58	
	others	6	1.03	5	1.47	4	1.22	2	3.77	13	1.7	
Number of family members		2.08	1.24	2.16	1.11	2.03	1.20	2.26	1.42	2.19	1.14	11.2670 *
Number of diagnosed diseases		4.73	1.99	4.97	2.20	4.77	1.96	4.79	2.07	5.05	2.25	14.9228 **
Dementia	Yes	306	52.40	183	53.82	177	54.13	35	66.04	396	51.83	4.3783
	No	278	47.60	157	46.18	150.00	45.87	18	33.96	368	48.17	
LTC grade	Grade 1	16	2.74	13	3.82	4	1.22	1	1.89	47	6.15	42.1947 **
	Grade 2	38	6.51	30	8.82	18	5.5	2	3.77	68	8.9	
	Grade 3	159	27.23	86	25.29	79	24.16	13	24.53	202	26.44	
	Grade 4	278	47.6	175	51.47	168	51.38	27	50.94	356	46.6	
	Grade 5	84	14.38	33	9.71	56	17.13	10	18.87	85	11.13	
	Grade 6	9	1.54	3	0.88	2	0.61	0	0	6	0.79	
Home care usage		1586.76	1695.21	1613.23	1479.10	1657.16	1728.52	1783.58	1647.89	1664.44	1690.89	10.4674 *
Region	Large city	178	30.48	130	38.24	77	23.55	9	16.98	277	36.26	37.5109 ***
	Medium and small city	174	29.79	93	27.35	108	33.03	15	28.3	244	31.94	
	Rural area	232	39.73	117	34.41	142	43.43	29	54.72	243	31.81	
Out-of-pocket money		157.20	108.69	119.40	102.92	152.12	108.34	115.94	79.95	127.57	109.30	9.3722
Age of caregiver		54.72	7.87	61.45	9.57	59.36	9.01	58.43	8.95	66.01	12.16	132.3913 ***
Care burden		2.69	0.94	2.73	1.12	2.50	0.96	2.62	0.93	2.97	1.04	15.3327 **
Caregiving Intensity		2.68	0.94	2.74	1.11	2.50	0.97	2.64	0.92	2.77	1.05	16.7717 **
Household income (KRW 100,000)	10>	24	4.11	109	32.06	42	12.84	18	33.96	341	44.63	473.9878 ***
	10–20	88	15.07	115.00	34	74	22.63	6.00	11.32	152	19.9	
	20–30	165	28.25	53	15.59	63	19.27	3	5.66	119	15.58	
	30–40	121	20.72	32	9.41	53	16.21	12	22.64	75	9.82	
	40–50	89	15.24	14	4.12	49	14.98	8	15.09	37	4.84	
	50–60	51	8.73	12	3.53	23	7.03	5	9.43	18	2.36	
	60<	46	7.88	5	1.47	23	7.03	1	1.89	22	2.88	
Relation with care recipients	Spouse	28	4.79	79	23.24	30	9.17	4	7.55	254	33.25	285.1716 ***
	Son	236	40.41	146	42.94	113	34.56	19	35.85	262	34.29	
	Daughter	65	11.13	33	9.71	33	10.09	14	26.42	87	11.39	
	Daughter-in-law	232	39.73	71	20.88	138	42.2	12	22.64	124	16.23	
	Others	23	3.94	11	3.24	13	3.98	4	7.55	37	4.84	

* *p* < 0.05; ** *p* < 0.01; *** *p* < 0.001.

**Table 6:** Factors affecting employment status of family caregivers (n = 2068).

Ref. Full-Time Employment		Part-Time and Temporary			Self-Employed			Unpaid Family Work			Unemployed		
		β	SE	RRR	β	SE	RRR	β	SE	RRR	β	SE	RRR
Female care recipient		−0.361	0.209	0.697	0.384	0.239	1.468	0.420	0.462	1.522	0.121	0.194	1.128
Having spouse		0.046	0.324	1.047	0.415	0.312	1.514	0.032	0.634	1.032	0.080	0.314	1.084
Ref. living alone	W/spouse	−0.324	0.395	0.724	−0.016	0.379	0.984	0.494	0.716	1.639	−0.257	0.377	0.774
	W/children	0.089	0.351	1.093	0.023	0.317	1.023	0.856	0.679	2.354	−0.040	0.324	0.961
	W/child + grandchild	0.043	0.489	1.044	−0.122	0.456	0.886	0.340	0.924	1.404	−0.096	0.455	0.908
	others	0.136	0.845	1.146	0.206	0.751	1.229	1.275	0.939	3.579	−0.123	0.733	0.884
LTC grade		0.020	0.096	1.021	0.153	0.099	1.165	0.321	0.179	1.379	−0.049	0.086	0.952
Home care usage		0.000	0.000	1.000	0.000	0.000	1.000	0.000	0.000	1.000	0.000	0.000	1.000
Ref. Large city	Medium and small city	−0.275	0.216	0.760	0.307	0.223	1.359	0.546	0.483	1.727	−0.215	0.192	0.807
	Rural area	−0.274	0.210	0.760	0.319	0.217	1.376	0.892	0.465	2.439	−0.364	0.190	0.695
Living with care recipients		0.613	0.338	1.845	0.285	0.315	1.329	0.136	0.584	1.146	0.552	0.320	1.737
Age of caregiver		0.053 ***	0.012	1.054	0.068 ***	0.011	1.070	0.038 *	0.020	1.039	0.092 ***	0.011	1.096
Household income		−0.468 ***	0.061	0.626	−0.005	0.048	0.995	−0.213 *	0.097	0.808	−0.406 ***	0.051	0.666
Care burden		0.048	0.088	1.049	−0.174 *	0.082	0.841	0.020	0.135	1.020	0.091	0.075	1.095
Caregiving Intensity		−0.003	0.085	0.997	−0.118	0.074	0.889	0.229	0.172	1.257	−0.142	0.075	0.867
Relation to care recipient (ref. son)	Spouse	0.669	0.496	1.953	−0.721	0.468	0.486	0.456	1.070	1.578	0.759	0.444	2.135
	Daughter	0.983 ***	0.213	2.673	−0.004	0.192	0.996	1.008 *	0.424	2.739	0.892 ***	0.189	2.439
	Daughter−in−law	1.017 **	0.306	2.764	0.054	0.279	1.056	1.831 ***	0.463	6.240	1.356 ***	0.255	3.880
	Others	0.064	0.577	1.066	0.328	0.463	1.389	1.406 *	0.661	4.080	1.135 ***	0.435	3.112
Cons.		−2.859 ***	0.919	0.057	−4.927 ***	0.974	0.007	−8.457 ***	1.819	0.000	−4.483 ***	0.844	0.011
		Wald Chi^2^ (76) = 505.47 ***/Pseudo R^2^ = 0.1395											

* *p* < 0.05; ** *p* < 0.01; *** *p* < 0.001.

## Data Availability

Details and downloads of data used in the study can be found at the following address. URL of datasets analyzed: (https://mdis.kostat.go.kr/ofrData/selectOfrDataDetail.do?survId=1004220&itmDiv=2&nPage=3&itemId=2007&itemNm=) (accessed on 4 March 2024).

## Public Involvement Statement

There was no public involvement in any aspect of this research.

## Guidelines and Standards Statement

This manuscript was drafted against the STROBE: The Strengthening the Reporting of Observational studies in Epidemiology.
